# Sonography in Hypotension and Cardiac Arrest (SHoC): Rates of Abnormal Findings in Undifferentiated Hypotension and During Cardiac Arrest as a Basis for Consensus on a Hierarchical Point of Care Ultrasound Protocol

**DOI:** 10.7759/cureus.564

**Published:** 2016-04-08

**Authors:** James Milne, Paul Atkinson, David Lewis, Jacqueline Fraser, Laura Diegelmann, Paul Olszynski, Melanie Stander, Hein Lamprecht

**Affiliations:** 1 Family Medicine Residency Program, Sydney, Nova Scotia, Dalhousie; 2 Emergency Medicine, Saint John Regional Hospital; 3 Emergency Medicine, Dalhousie University; 4 Emergency Medicine, Horizon Health Network; 5 Emergency Medicine, University of Maryland; 6 Emergency Medicine, Saskatoon Health Region; 7 Division of Emergency Medicine, University of Cape Town; 8 Emergency Medicine, Stellenbosch University

**Keywords:** ultrasound, echocardiography, shock, advanced cardiac life support

## Abstract

**Introduction:**

Point of care ultrasound (PoCUS) has become an established tool in the initial management of patients with undifferentiated hypotension. Current established protocols (RUSH and ACES) were developed by expert user opinion, rather than objective, prospective data. PoCUS also provides invaluable information during resuscitation efforts in cardiac arrest by determining presence/absence of cardiac activity and identifying reversible causes such as pericardial tamponade. There is no agreed guideline on how to safely and effectively incorporate PoCUS into the advanced cardiac life support (ACLS) algorithm. We wished to report disease incidence as a basis to develop a hierarchical approach to PoCUS in hypotension and during cardiac arrest.

**Methods:**

We summarized the recorded incidence of PoCUS findings from the initial cohort during the interim analysis of two prospective studies. We propose that this will form the basis for developing a modified Delphi approach incorporating this data to obtain the input of a panel of international experts associated with five professional organizations led by the International Federation of Emergency Medicine (IFEM). The modified Delphi tool will be developed to reach an international consensus on how to integrate PoCUS for hypotensive emergency department patients as well as into cardiac arrest algorithms.

**Results:**

Rates of abnormal PoCUS findings from 151 patients with undifferentiated hypotension included left ventricular dynamic changes (43%), IVC abnormalities (27%), pericardial effusion (16%), and pleural fluid (8%). Abdominal pathology was rare (fluid 5%, AAA 2%). During cardiac arrest there were no pericardial effusions, however abnormalities of ventricular contraction (45%) and valvular motion (39%) were common among the 43 patients included.

**Conclusions:**

A prospectively collected disease incidence-based hierarchy of scanning can be developed based on the reported findings. This will inform an international consensus process towards the development of proposed SHoC protocols for hypotension and cardiac arrest, comprised of the stepwise clinical-indication based approach of Core, Supplementary, and Additional PoCUS views. We hope that such a protocol would be structured in a way that enables the clinician to only perform views that are clinically indicated, which limits exposure to the frequent incidental positive findings that accompany the current “one size fits all” standard protocols.

## Introduction

The use of point of care ultrasound (PoCUS) as an adjunct to the practice of emergency medicine is now well established internationally. Initially, evidence to support the use of PoCUS came from the management of blunt trauma patients [1]. The scope of practice has expanded as emergency physicians have identified further clinical problems where PoCUS is able to aid diagnosis and guide procedures.  Assessment of patients in cardiac arrest and with undifferentiated hypotension have been core applications of PoCUS, with various protocols in widespread use in emergency medicine [2-3]. Such protocols are based upon a logical approach to identifying the likely etiology and guiding therapy, but these are frequently based solely on expert opinion and not on the actual incidence of disease. They have become essential components of the initial investigation for patients presenting with undifferentiated hypotension, as they enable clinicians to quickly and accurately determine the source of shock [4]. In this paper we present the incidence of positive findings that we hope will inform the development of a Sonography in Hypotension and Cardiac Arrest (SHoC) Protocol so that clinicians may safely incorporate PoCUS into the resuscitation of the hypotensive or arrested patient. To initiate development of this protocol, we report the incidence of PoCUS findings from the interim analyses of two ongoing prospective studies involving emergency department patients in cardiac arrest or with undifferentiated hypotension. These observed incidences will provide insight into the value of each specific ultrasound view. We plan that the protocol will be further developed by a structured review process involving a panel of 24 international experts associated with five international professional organizations, led by the International Federation for Emergency Medicine (IFEM) ultrasound curriculum group.

## Materials and methods

We reviewed and report all findings from the interim analyses of two multicenter prospective studies. These studies examined ultrasound use in undifferentiated hypotension and in cardiac arrest. In each study, comprehensive point of care ultrasound protocols were completed. The interim analyses were scheduled for a recruitment point of 150 patients in first Sonography in Hypotension and Cardiac Arrest in the Emergency Department (SHoC-ED1) study (Research Ethics Board Approval Number HHN RS 2011-1590; unpublished data) and on completion of local data collection in the Sonography in cardiac arrest: Real-time Assessment and Evaluation with Sonography – Outcomes Network (REASON) study (Research Ethics Board Approval Number HHN RS#2011-1566; unpublished data).

## Results

Rates of abnormal PoCUS findings in 82 patients from the first included 151 SHoC-ED1 patients with undifferentiated hypotension are shown in Table 1 and consisted of cardiac, IVC and lung findings, including left ventricular dynamic changes (43%), IVC abnormalities (27%), pericardial effusion (16%), and pleural fluid (8%). Abdominal pathology was rare (fluid 5%, AAA 2%).

**Table 1:** International Data for Prevalence of Findings in Undifferentiated Hypotension (LV: left ventricle; IVC: inferior vena cava; AAA: abdominal aortic aneurysm)

Sonography in Hypotension	
Finding	Frequency
LV Dynamic Change	43%
IVC Abnormalities	27%
Pericardial Effusion	16%
Pleural Fluid	8%
Peritoneal Fluid	5%
AAA	2%

Data was analyzed for 43 patients who had sonography performed during cardiac arrest during the REASON study. Abnormal findings are summarized in Table 2. There were no pericardial effusions, however abnormalities of ventricular contraction (45%) and valvular motion (39%) were common.

**Table 2:** Local Data for Prevalence of Findings in Cardiac Arrest

Sonography in Cardiac Arrest	
Finding	Frequency
Contractility Abnormality	45%
Abnormal Valve Function	39%
Pericardial Effusion	0%

## Discussion

The rate of findings from these studies indicates that most positive finds relate to cardiac, lung, and IVC scanning. This data shows that there may be a low yield for certain previously suggested components of protocols, including aorta and abdominal scanning. We hope to provide these results to a panel of experts to be used, along with their combined clinical expertise, to identify what role a given view should play in the proposed SHoC protocol. This panel will be asked to consider the evidence they were provided and to vote in a way that would yield an efficient protocol consistent with current PoCUS practice and resuscitation science. When deriving this protocol, the SHoC team identified an international trend in the approach to teaching PoCUS. This trend recognizes four features that any protocol must identify to be able to adequately assess a person’s cardiovascular status. By ensuring that the SHoC protocol has the same foundations as the current approach to teaching ultrasound internationally, we hope to maximize clinician familiarity with our protocol. The features that constitute the “4F” approach are described in Table 3.

**Table 3:** Proposed Approach to Purpose of Scans for the Proposed SHoC Protocol. (LV: left ventricle; RV: right ventricle; IVC: inferior vena cava)

Cardiac Views	
1. Fluid	Is there a significant pericardial effusion?
2. Form	Is the heart small, normal or large? Is the LV larger than the RV?
3. Function	Is there vigorous contractility? Are the valves opening?
IVC and Lung Views	
4. Filling	Is the IVC dilated and non-collapsing? Is the IVC small and collapsing? Are there multiple B-lines in the lungs bilaterally? Is there pleural fluid?

The SHoC derivation team will examine the literature and will consult our expert panel to identify views that may be valuable in identifying aetiology of undifferentiated hypotension and which views are a priority in cardiac arrest patients. To reach an unbiased consensus, the Delphi Method will be implemented [5].

### The Delphi method

The Delphi method is a consensus-building tool that has been used in many fields (medical and otherwise) to enable a group to arrive at a “round table” conclusion. This is done using a central non-voting coordinator who identifies a set of “issues” the panel must address. For each issue, a list of discrete possible options is provided to the expert panel. The experts then answer the “issues” with one of the provided responses, and also provide a rationale for their decision. The central coordinator then reviews the panel’s responses. If a consensus about a certain issue is reached, that issue does not need to be included in future iterations of the Delphi method. If, however, consensus is not reached for an issue, the central coordinator examines the rationales provided by the experts, and collates these into “opinion summaries”. Options that are unpopular and therefore unlikely to develop into consensus are eliminated from future rounds. The remaining options, as well as the opinion summaries and the frequencies with which those options were selected are then again presented to the panel. The process then repeats in the next iteration. This continues until consensus is built, and reasonable options are found for all issues. We plan to develop a hierarchical approach to PoCUS in hypotension and cardiac arrest using the categories outlined in Table 4.

**Table 4:** Prioritization of Views for Development of the SHoC Protocol

Prioritization of View for Development	
1. Core	To be performed routinely for ALL patients.
2. Supplementary	To be performed for all patients where this would likely add further information without delaying ongoing critical care.
3. Additional	To be performed when clinically indicated according to the specific clinical circumstances.
4. Do not include	Not appropriate for patients with undifferentiated hypotension or cardiac arrest.

## Conclusions

We present an approach to develop an international consensus for the use of sonography in hypotension and cardiac arrest (SHoC) based on prospectively collected disease incidence. The proposed SHoC protocols for hypotension and cardiac arrest would be stepwise clinical-indication based approaches, incorporating core, supplementary, and additional PoCUS views focusing on scanning for fluid, form, function and filling.
